# *Tppp3*^*+*^ synovial/tendon sheath progenitor cells contribute to heterotopic bone after trauma

**DOI:** 10.1038/s41413-023-00272-x

**Published:** 2023-07-21

**Authors:** Ji-Hye Yea, Mario Gomez-Salazar, Sharon Onggo, Zhao Li, Neelima Thottappillil, Masnsen Cherief, Stefano Negri, Xin Xing, Qizhi Qin, Robert Joel Tower, Chen-Ming Fan, Benjamin Levi, Aaron W. James

**Affiliations:** 1 Department of Pathology, Johns Hopkins University, Baltimore, MD 21205 USA; 2 Orthopaedic and Trauma Surgery Unit, Department of Surgery, Dentistry, Paediatrics and Gynaecology of the University of Verona, Verona, Italy; 3 Center for Organogenesis and Trauma, Department of Surgery, University of Texas Southwestern, Dallas, TX USA; 4 Carnegie Institution for Science, Baltimore, MD USA

**Keywords:** Bone, Pathogenesis

## Abstract

Heterotopic ossification (HO) is a pathological process resulting in aberrant bone formation and often involves synovial lined tissues. During this process, mesenchymal progenitor cells undergo endochondral ossification. Nonetheless, the specific cell phenotypes and mechanisms driving this process are not well understood, in part due to the high degree of heterogeneity of the progenitor cells involved. Here, using a combination of lineage tracing and single-cell RNA sequencing (scRNA-seq), we investigated the extent to which synovial/tendon sheath progenitor cells contribute to heterotopic bone formation. For this purpose, *Tppp3* (tubulin polymerization-promoting protein family member 3)-inducible reporter mice were used in combination with either *Scx* (Scleraxis) or *Pdgfra* (platelet derived growth factor receptor alpha) reporter mice. Both tendon injury- and arthroplasty-induced mouse experimental HO models were utilized. ScRNA-seq of tendon-associated traumatic HO suggested that *Tppp3* is an early progenitor cell marker for either tendon or osteochondral cells. Upon HO induction, *Tppp3* reporter^*+*^ cells expanded in number and partially contributed to cartilage and bone formation in either tendon- or joint-associated HO. In double reporter animals, both *Pdgfra*^*+*^*Tppp3*^*+*^ and *Pdgfra*^*+*^*Tppp3*^*-*^ progenitor cells gave rise to HO-associated cartilage. Finally, analysis of human samples showed a substantial population of TPPP3-expressing cells overlapping with osteogenic markers in areas of heterotopic bone. Overall, these data demonstrate that synovial/tendon sheath progenitor cells undergo aberrant osteochondral differentiation and contribute to HO after trauma.

## Introduction

Heterotopic ossification (HO) is a pathological process involving the formation of heterotopic endochondral and intramembranous bone in muscles, tendons, ligaments, and other soft tissues.^1–3^ HO occurs at a higher frequency with certain conditions, including traumatic tissue injury such as arthroplasty, fractures, and severe burns.^4–6^ HO after hip arthroplasty is particularly common and is thought to be induced by trauma and local inflammation. However, HO induced by burns may be triggered by a combination of factors, including local trauma tissue damage and systemic inflammation.^4^ Ultimately, HO formation is characterized by abnormal differentiation of tissue resident mesenchymal progenitor cells undergoing endochondral and intramembranous ossification^7,8^ and occurs at high frequency in synovial lined tissues such as around joints and in tendoligamentous tissues.^4,9^

Several studies have examined which specific mesenchymal progenitor subsets are the main contributors to HO and have used reporter mice to assess *Prx1*,^10^ *Pdgfra*,^11^ *Dermo1*,^12^ *Mx1*,^13^ *Scx* and *Gli1*.^14^ One limitation of these reporter animals is that these molecules are generally expressed by a wide variety of cell types. For instance, *Pdgfra* is a canonical mesenchymal progenitor cell marker^15^ expressed in many cells involved in tissue healing, indicating it is a nonspecific marker. Indeed, a detailed cellular layout of the heterogeneous mesenchymal progenitor cell population would substantially improve our understanding of aberrant stem cell differentiation during HO. Recently, a site-specific HO progenitor was identified using the Hoxa11-CreER lineage tracing system.^11^ However, further elucidation of the subpopulations of HO progenitors in different tissue types is necessary. One crucial stem cell niche in the tendon is the peritenon, where tendon stem/progenitor cells (TSPCs) reside.^16^ For example, Nestin^+^ TSPCs reside in the peritenon in the perivascular areas,^17^ which may correlate with another tendon progenitor cell expressing CD146.^18^ More recently, tubulin polymerization-promoting protein family member 3 (*Tppp3*)-expressing cells have been identified as tendon sheath/peritenon progenitor cells that contribute to tendon repair.^19^ This study demonstrated the progenitor potential of *Tppp3*^+^ TSPCs and the different cell fates of these progenitors depending on the coexpression of *Pdgfra*-segregating tendon progenitors from fibro-adipogenic progenitor cells.^19^ Extending these findings, we investigated how *Tppp3*^+^ TPSCs and other cells may contribute to aberrant cell differentiation during HO.

Here, we utilized a combination of *Tppp3* inducible reporter mice and single-cell RNA sequencing analysis to determine the extent to which *Tppp3*^+^ synovial/tendon sheath progenitors are involved in HO formation. Two previously validated HO trauma models were utilized: an Achilles tendon HO model and a hip postarthroplasty HO model.^1,20^ After arthroplasty surgery, HO can occur in up to 40% of patients.

Briefly, *Tppp3*^+^ cells are restricted to the synovial membranes, a specialized type of connective tissue around tendons such as the tendon sheath, which upon traumatic injury prominently expand in number and were found to partially contribute to HO. Importantly, transcriptomic evidence suggests that *Tppp3*^*+*^ cells not only represent primitive mesenchymal progenitor cells but may also have regulatory effects on HO formation by releasing soluble molecules that promote osteogenic differentiation.

## Results

### *Tppp3*^+^ progenitor cells in the tendon sheath expand after HO induction in the Achilles tendon

**Fig. 1:**
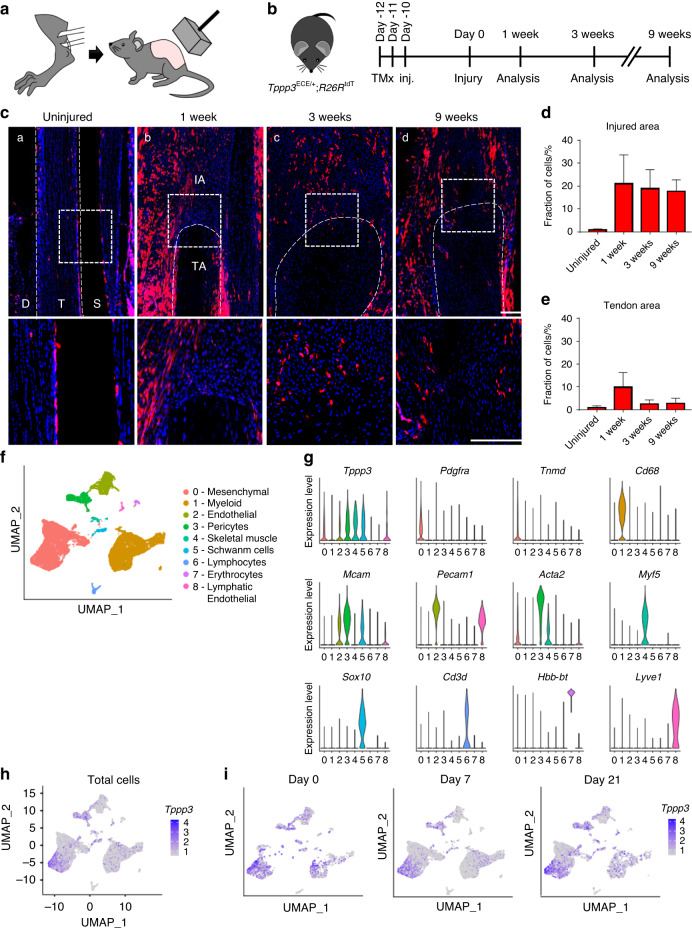
Tppp3^+^ tendon sheath progenitors expand at the heterotopic ossification (HO) induction site after Achilles tendon injury. **a** Schematic representation of HO induction, including complete Achilles tenotomy (left) and dorsal burn (right). **b** *Tppp3*^ECE/+^;R26R^tdT^ animals were administered tamoxifen (TMx) for three continuous days, followed by a 10-day washout period before HO induction. Reporter activity was examined at 1, 3 and 9 weeks after injury. **c** tdT^+^ (*Tppp3*) cells (red) in the defect area were visualized using sagittal sections of the distal tenotomy site after injury. tdT^+^ cells were present outside of the tendon (epitenon) in the uninjured condition, and the cells expanded into the defect area of the tendon after injury. The dashed white line indicates the margins of the Achilles tendon, and the dashed white box in the upper panels is magnified in the lower panels (scale bars: 200 µm). D: deep; T: tendon; S: superficial, IA: injured area, TA: tendon area. **d** Fraction of tdT^+^ cells in the injured area. **e** Fraction of tdT^+^ cells in the residual tendon area (under dashed white line). **f** UMAP visualization of cell clusters from the HO induction site at 0, 7, and 21 d post-injury. **g** Violin plots showing the expression of markers for cell type identification. **h** UMAP of *Tppp3* expression across all clusters (all time points together). **i** UMAP showing the expression of *Tppp3* in cell clusters across time points. *n* = 3 animals per timepoint for histology; for scRNA-seq, *n* = 4 animals per timepoint; 3 678, 13 358 and 5 366 cells at timepoints 0, 7 and 21 d, respectively

To investigate the contribution of *Tppp3*^*+*^ cells to heterotopic ossification, we first used our validated model of burn/tenotomy to induce traumatic HO in *Tppp3*^ECE/*+*^*;R26R*^tdT^ (*Tppp3*-tdT) reporter mice, which have a knock-in allele of ER^T2^ (Fig. 1a, b). After HO induction was performed in the Achilles tendon, samples were harvested at 0, 1, 3 and 9 weeks corresponding to the uninjured, fibroproliferative, chondrogenic and osteogenic phases of HO, respectively (Fig. 1b). In uninjured conditions, 87% of Tppp3-immunoreactive cells showed tdT^+^ expression in the Achilles tendon (Fig. S1). Moreover, *Tppp3*-tdT^+^ cells had limited distribution, primarily in the tendon sheath, and minimal activity within the tendon body (Fig. 1c; a and Fig. S2). Characterization of FACS-isolated *Tppp3*-tdT^+^ cells demonstrated multipotentiality, including adipogenic, osteogenic, chondrogenic and tenogenic differentiation potential (Fig. S3). After HO-induced injury, a rapid increase in the number of tdT^+^ cells was observed in the injury site at 1 wk (21.09% of the injury area) (Fig. 1c; a′, b′, d). Cells marked by tdT persisted within the injury site across all timepoints but were slightly reduced at both 3 and 9 weeks (18.93% and 17.76% of HO site cells at 3 and 9 weeks, Fig. 1c; c′, d′, d). However, the cells within the tendon area (uninjured area) slightly increased at 1 week and decreased at 3 weeks (Fig. 1e).

**Fig. 2:**
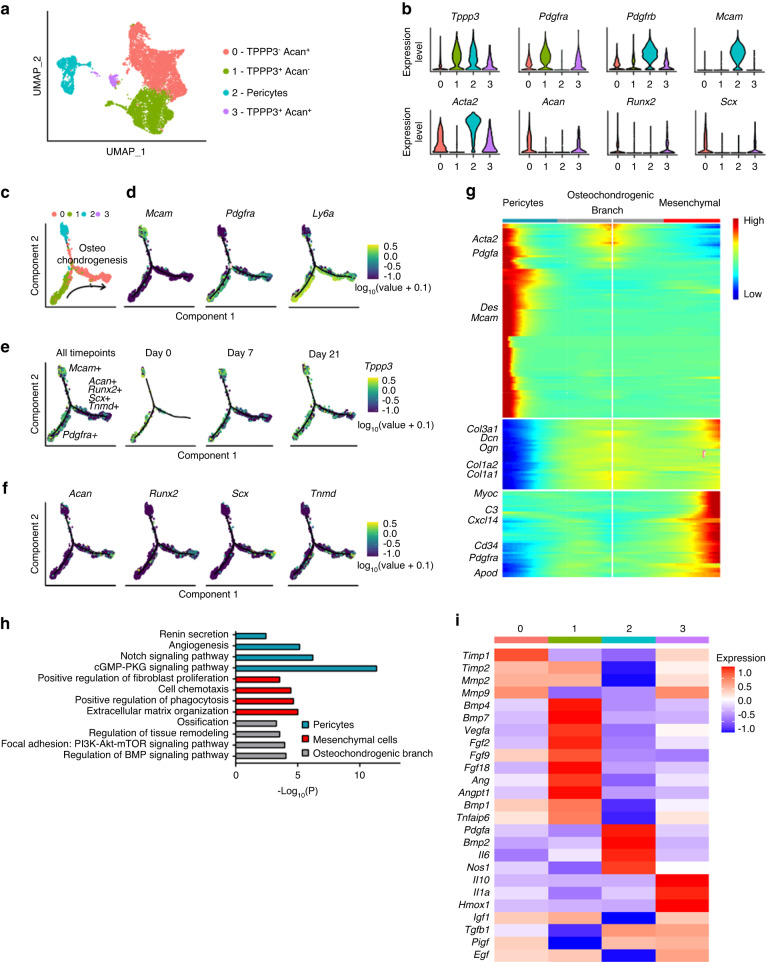
Cell subclustering and pseudotemporal trajectory analysis reveal a hierarchy of osteochondrogenic progenitors in relation to Tppp3 expression. **a** UMAP visualization of mesenchymal and pericyte subclusters. Three subclusters were found among mesenchymal cells: Cluster 0 (*Tppp3*^-^ *Acan*^+^), Cluster 1 (*Tppp3*^+^ *Acan*^-^) and Cluster 3 (*Tppp3*^+^ *Acan*^+^). **b** Expression of mesenchymal (*Pdgfra*), pericyte (*Pdgfrb, Mcam, Acta2*) and tenocyte-like cells (*Scx*) and osteochondrogenic markers (*Runx2, Acan*) in each cell subcluster. **c** Pseudotime trajectory analysis of the four subclusters. **d** Expression of pericyte and mesenchymal markers across pseudotime. **e** Expression of *Tppp3* across pseudotimes at all timepoints or individually at each time point. **f** Expression of tenocyte (*Scx, Tnmd*), chondrocyte (*Acan)* and osteoblast-related genes (*Runx2*) across pseudotime. **g** Heatmap of the pseudotime trajectory of pericytes (left), mesenchymal cells (right), and their common osteochondrogenic branch (center). **h** GO term analysis of clusters identified with pseudotime analysis, including pericytes, mesenchymal cells, and osteochondrogenic cells. **i** Heatmap showing the expression of secreted factors that regulate heterotopic ossification in the four cell subclusters. *n* = 4 biological replicates per time point. Animals and 1 460 (0 d), 6 317 (7 d) and 3 484 (21 d) mesenchymal cells per timepoint for scRNA-seq

To better understand the cellular dynamics upon HO induction, we analyzed our previously published scRNA-seq dataset of Achilles tendon HO^20^ in which 9 cell clusters were identified based on the expression of known markers (Fig. 1f, g). In particular, we focused on the expression profile of *Tppp3* in uninjured tendons and at 7 and 21 d post-injury (Fig. 1h, i). We found that *Tppp3* is expressed primarily by mesenchymal cells, as well as several smaller cell clusters, such as pericytes, skeletal muscle cells, and Schwann cells (Fig. 1g–i). However, upon HO induction, the expression of *Tppp3* was increased in the mesenchymal clusters, accompanied by an expansion of this cell cluster (Fig. 1l). Next, the mesenchymal and pericyte cell clusters were subsetted, reintegrated, and further stratified into 4 clusters based on the expression of *Tppp3* and Aggrecan (*Acan*) to follow the cartilage formation phase of endochondral ossification (Fig. 2a). The expression of canonical mesenchymal (*Pdgfra*) and pericyte markers (*Pdgfrb, Mcam, Acta2*) corroborated their identity, and variable expression of *Tppp3* was observed among clusters (Fig. 2b). To further determine the hierarchy of these cell subclusters, we performed pseudotime trajectory analysis (Fig. 2c), showing that Cluster 1 (*Tppp3*^+^*Acan*^-^) went into a common branch toward a differentiated state where Clusters 0 and 3 were overrepresented (Fig. 2d). Analysis of the expression of *Tppp3* over pseudotime showed that *Tppp3* is present in early pseudotime among the different time points (Fig. 2e), again indicating its expression in progenitor cells. The expression of tendon (*Scx, Acan, Tnmd*) and bone-related markers (*Runx2*) was higher in late pseudotime (Fig. 2f). Altogether, our data support a primitive/progenitor cell phenotype for *Tppp3*-expressing cells, which is reduced upon differentiation.

Subsequently, we performed analysis of differential expression across pseudotime between pericytes, mesenchymal cells, and the common branch, named here “osteochondrogenic” (Fig. 2g). The expression of *Acta2*, *Mcam, Pdgfa* and *Des* was enriched in pericytes, among other genes. However, mesenchymal cells expressed higher levels of *Cd34, Pdgfra, Apod* and *Cxcl14*. Next, with the differentially expressed genes in every cluster across pseudotime, GO term analysis was performed (Fig. 2h), which showed enrichment of renin secretion and angiogenesis in pericytes, whereas in mesenchymal cells, the main processes overrepresented were extracellular matrix organization and fibroblast proliferation. In the case of the osteochondrogenic branch, GO terms related to ossification, tissue remodeling and BMP signaling were enriched.

Finally, analysis of the expression of secreted ligands of molecules reported to promote osteochondrogenic differentiation in HO and other biological processes was performed among the cell clusters (Fig. 2l). The majority of the pro-ossification ligands (such as *Bmp4, Bmp7, Vegfa, Fgf2, Fgf9* and *Fgf18*) were expressed in Cluster 1, which is high in *Tppp3* and low in *Acan* expression, suggesting a regulatory role. Pericytes also expressed genes that promote ossification, such as *Bmp2* and *Pdgfa*. However, Clusters 0 and 3 expressed other molecules, such as *Timp1, Mmp2, Mmp9, Il-10* and *Il-1a*, to a lesser degree. Overall, these data indicate that Cluster 1 is more involved in the regulation of ossification than the more differentiated clusters, thus suggesting a regulatory role in HO formation among some *Tppp3*^+^ cells.

### *Tppp3*^+^ cells contribute to heterotopic cartilage formation after HO induction in the Achilles tendon

**Fig. 3:**
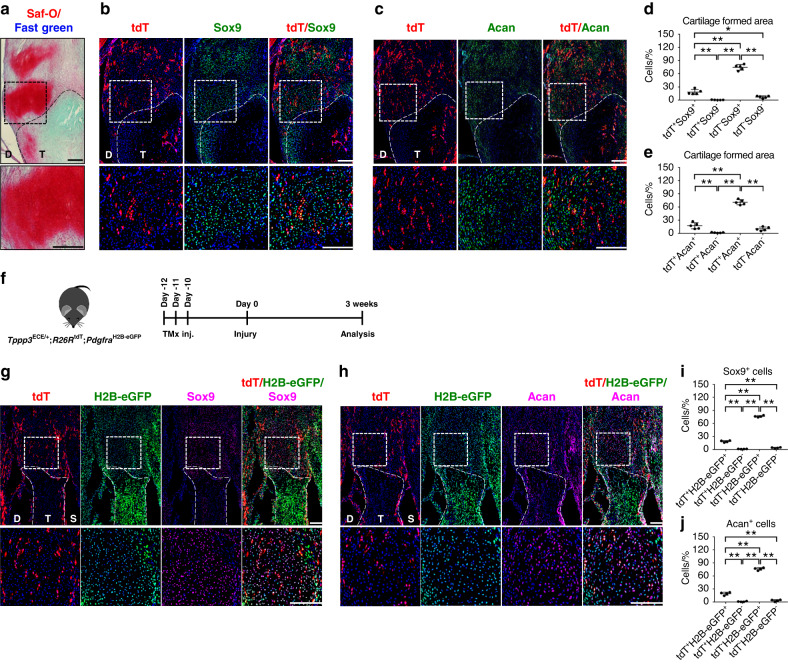
Tppp3^+^ cells give rise to cartilage after HO induction in the Achilles tendon. **a** Cartilage area was visualized using Saf-O/Fast Green staining. Cartilage-like matrix appears red. The dashed black line indicates the margins of the tendon. The dashed black box in the upper panels is magnified in the lower panels (scale bars in panels: 200 µm). D, deep; T, tendon. **b**, **c** tdT^+^ cells and Sox9 or Acan immunohistochemical staining within representative sagittal sections of the distal tenotomy site. The dashed white line indicates the margins of the remaining tendon tissue. The dashed white box in the upper panels is magnified in the lower panels (scale bars in panels: 200 µm). D: deep; T: tendon. **d**, **e** Quantification of tdT^+^ and Sox9^+^ or Acan^+^ cells within the cartilage-formed area (*n* = 5 animals per group). **f** Schematic of the experiment: *Tppp3*^ECE/+^;*R26R*^tdT^;*Pdgfra*^H2B-eGFP^ animals were administered TMx for three continuous days, and reporter activity was examined at 3 weeks after injury. **g**, **h** tdT^+^ and H2B-eGFP (*Pgdfra*) ^+^ cells and Sox9 or Acan immunohistochemical staining within representative sagittal sections of the distal tenotomy site. The dashed white line indicates the margins of the remaining tendon tissue. The dashed white box in the upper panels is magnified in the lower panels (scale bars in panels: 200 µm). D, deep side; T, tendon area; S, superficial side. **i**, **j** Quantification of tdT^+^ and H2B-eGFP^+^ Sox9^−^ or Acan^−^ expressing cells within the cartilage-formed area (*n* = 4 animals per group). For all graphs, each dot represents a single animal, with the mean ± 1 SD indicated. Statistical analysis was performed using one-way ANOVA with Tukey’s post hoc test. ^*^P < 0.05 and ^**^P < 0.01

To investigate the contribution of *Tppp3*^+^ cells to the formation of heterotopic cartilage matrix during HO, we analyzed *Tppp3*-tdT reporter mice at 3 weeks after HO-inducing injury. Saf-O/fast-green staining showed cartilage formation in the region of interest (Fig. 3a). Immunofluorescence staining was then used to corroborate the expression of cartilage markers. Indeed, Sox9 and Acan showed positive expression in the injured tissue area, overlapping with our previous histological staining results. Moreover, coexpression of *Tppp3*-tdT and cartilage markers was observed, which demonstrated that *Tppp3*^+^ cells accounted for 20.58% and 18.70% of Sox9^+^ and Acan^+^ cells, respectively (Fig. 3b–e).

Notably, only one-fifth of cartilage cells (Sox9^+^/Acan^+^) were descendants of *Tppp3*^+^ cells, suggesting a diverse cellular origin during endochondral ossification. To further investigate the type of *Tppp3*^+^ cells in our model, we crossed *Tppp3-tdT* reporter mice with constitutive *Pdgfra*-GFP reporter mice (Fig. 3f) to corroborate the mesenchymal origin of cells contributing to endochondral ossification. As expected, the majority of Sox9^+^ and Acan^+^ cells showed *Pdgfra* reporter activity (Fig. 3g, h) at 94.83% and 95.29%, respectively, and 20% of these *Pdgfra*-expressing cells showed *Tppp3*-tdT (Fig. 3i, j). These results demonstrate the mesenchymal origin of heterotopic cartilage formation and depict the cellular complexity of contributors to HO formation, in which *Tppp3*^+^ cells represent a subset of mesenchymal cells forming abnormal tissue.

### *Tppp3*^+^ cells contribute to bone formation after HO induction in the Achilles tendon

**Fig. 4:**
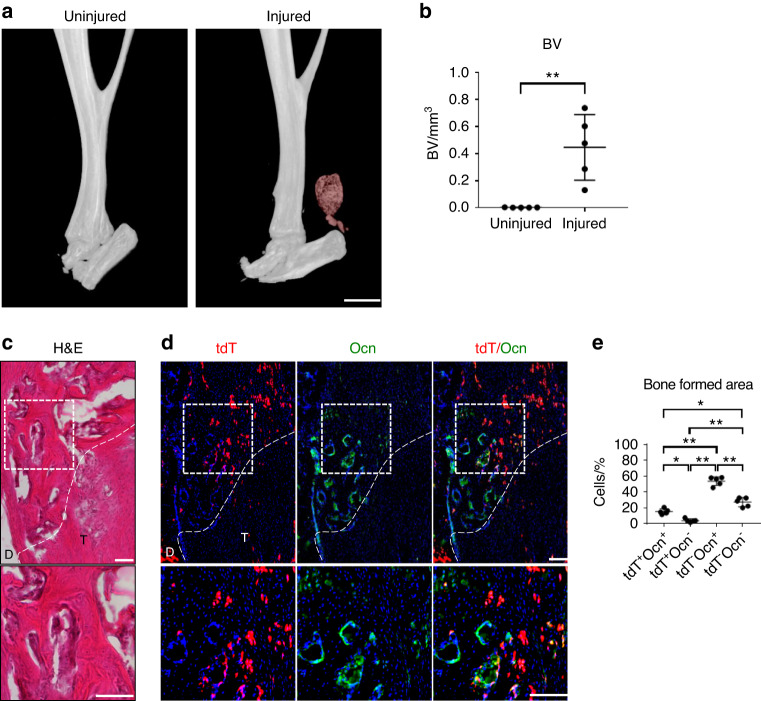
Tppp3^+^ cell contribution to bone areas within the HO induction site of the Achilles tendon. **a** µCT reconstruction of the injured Achilles tendon at 9 weeks post-injury. Heterotopic bone appears red, while native bone appears white (scale bar: 1 mm). **b** µCT quantification of heterotopic bone volume (BV) of the distal tenotomy site (*n* = 5 animals per group). **c** The bone formed area was visualized using H&E staining within representative sagittal sections of the distal tenotomy site. The dashed white line indicates the margins of the remaining tendon tissues. The dashed white box in the upper panels is magnified in the lower panels (scale bar in the upper panel: 200 µm, scale bar in the lower panel: 100 µm). **d** tdT^+^ cells and Ocn immunohistochemical staining within representative sagittal sections of the distal tenotomy site. The dashed white line indicates the margins of the tendon tissue. The dashed white box in the upper panels is magnified in the lower panels (scale bars in upper panels: 200 µm, scale bars in lower panels: 100 µm). **e** Quantification of tdT^+^ and Ocn^+^ cells within the bone-formed area (*n* = 5 animals per group). D: deep; T: tendon. For all graphs, each dot represents a single animal, with the mean ± 1 SD indicated. Statistical analysis for (**b**) was performed using two-tailed Student’s *t* test. Statistical analysis for (**e**) was performed using one-way ANOVA with Tukey’s post hoc test. ^*^P < 0.05 and ^**^P < 0.01

Next, the contribution of *Tppp3*+ cells to the osseous phase of HO was investigated. First, 3-D µCT reconstructions of a representative Achilles tendon showed abundant HO formation at 9 weeks after injury (Fig. 4a). All injured Achilles tendons had heterotopic ossification, and the mean volume of the newly formed bone was 0.44 mm^3^, whereas the uninjured Achilles tendon (contralateral side) showed no bone formation (Fig. 4a, b). Second, H&E staining showed the structure and the degree of aberrant bone formation within the tendon (Fig. 4c). Finally, immunofluorescence staining for the bone marker osteocalcin (OCN) was performed together with visualization of *Tppp3* reporter activity (Fig. 4d). The results showed that 23% of OCN-immunoreactive cells showed *Tppp3*^+^ reporter activity (Fig. 4d, e). These results showed that heterotopic cartilage at 3 weeks differentiated into bone at 9 weeks and that *Tppp3*^+^ cells contributed to a minority of the total osteoblasts within HO of the injured Achilles tendon.

### *Tppp3*^+^ cells contribute to tendon formation after HO induction in the Achilles tendon

**Fig. 5:**
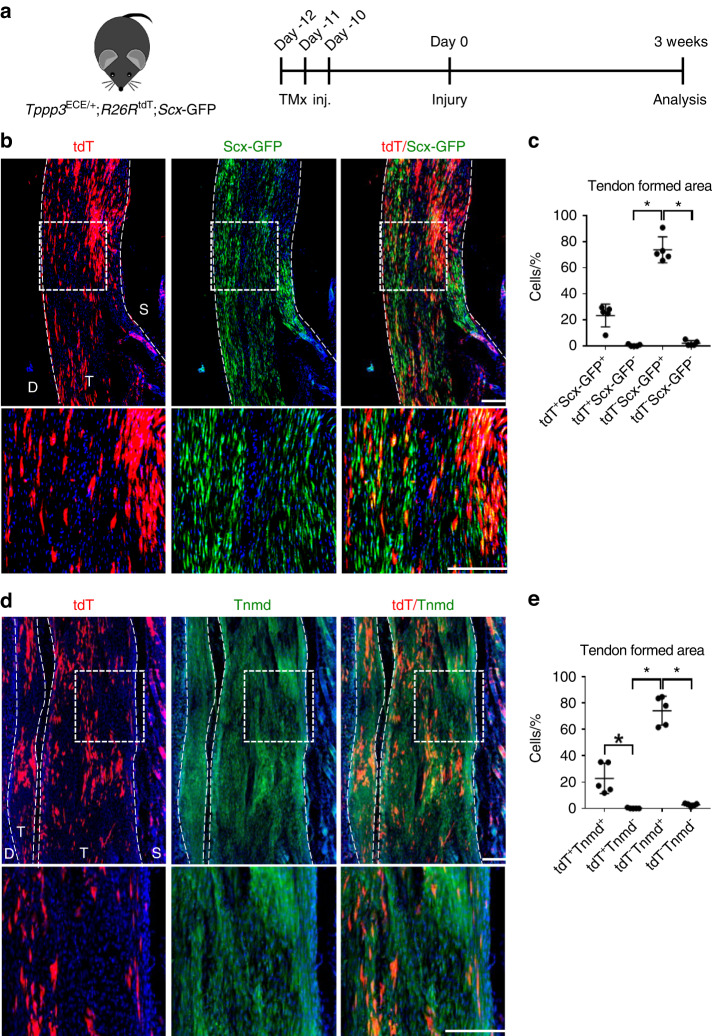
Tppp3^+^ cells give rise to a portion of tenocytes in the HO site. **a** Schematic of the experiment: *Tppp3*^ECE/+^;*R26R*^tdT^;*Scx*-GFP animals were administered TMx for 3 continuous d, and reporter activity was examined at 3 weeks after injury. **b** tdT^+^ and *Scx*-GFP expression within representative sagittal sections of the middle area of the tenotomy site. The dashed white line indicates the margins of tendon-like tissue. The dashed white box in the upper panels is magnified in the lower panels (scale bars in panels: 200 µm). **c** Quantification of tdT^+^ and Scx-GFP^+^ cells within the tendon-formed area (*n* = 5 animals per group). **d** tdT^+^ cells and Tnmd immunofluorescence staining within representative sagittal sections of the middle tenotomy site. The dashed white line indicates the margins of tendon-like tissue. The dashed white box in the upper panels is magnified in the lower panels. **e** Quantification of tdT^+^ and Tnmd^+^ cells within the tendon-formed area (*n* = 4 animals per group) (scale bars in panels: 200 µm). D: deep; T: tendon; S: superficial. For all graphs, each dot represents a single animal, with the mean ± 1 SD indicated. Statistical analysis was performed using the Kruskal‒Wallis test with Dunn’s post hoc multiple comparisons test. ^*^P < 0.05

As mentioned, Tppp3 is primarily considered a marker of tendon progenitor cells.^19,21^ In the context of HO formation, the role of *Tppp3*^*+*^ cells in tendon remodeling is unknown. Using inducible *Tppp3-tdT* reporter mice crossed with mice with the constitutive reporter for *Scx*,^19^ a tendon marker, we performed an analysis of the contribution of *Tppp3*^+^ cells during tendon remodeling (Fig. 5a). After HO-induced injury, a newly formed tendon-like matrix defined by the expression of *Scx*-GFP was observed in the tenotomy site at 3 weeks (Fig. 5b). A tendon-like matrix was formed most prominently at the middle aspect of the Achilles tendon defect, whereas heterotopic cartilage formation was formed closer to the ends of the residual tendon. Quantification showed that 23.27% of *Scx*-GFP cells within the injury area were descendants of *Tppp3*^+^ cells (Fig. 5c). Tenomodulin (TNMD) staining was also performed to validate the tendon phenotype of the newly formed tissue (Fig. 5d). Similar to *Scx*-GFP reporter activity, 24.47% of TNMD-immunoreactive cells showed *Tppp3* reporter activity (Fig. 5e). Given the above findings, we next returned to our scRNA-seq data and evaluated genes associated with the regulation of tenogenic differentiation among *Tppp3*-expressing and nonexpressing cell clusters (Fig. S4).^22^ Transcriptomic analysis showed that both Cluster 0 (*Tppp3*^-^, *Acan*^+^) and Cluster 1 (*Tppp3*^+^*Acan*^-^) expressed genes encoding soluble molecules that promote tendon differentiation, such as *Gdf5*, *Ctgf* and *Tgfb3* (Cluster 0), as well as *Gdf6*, *Fgf2* and *Tgfb2* (Cluster 1). These results implicate autocrine and paracrine signaling derived from *Tppp3*^*+*^ and other cells (*Tppp3*^*-*^) in the regulation of tendon repair versus HO formation within a traumatized microenvironment.

### *Tppp3*^+^ cells contribute to heterotopic cartilage formation at an intracapsular site after HO induction in a hip postarthroplasty HO model

**Fig. 6:**
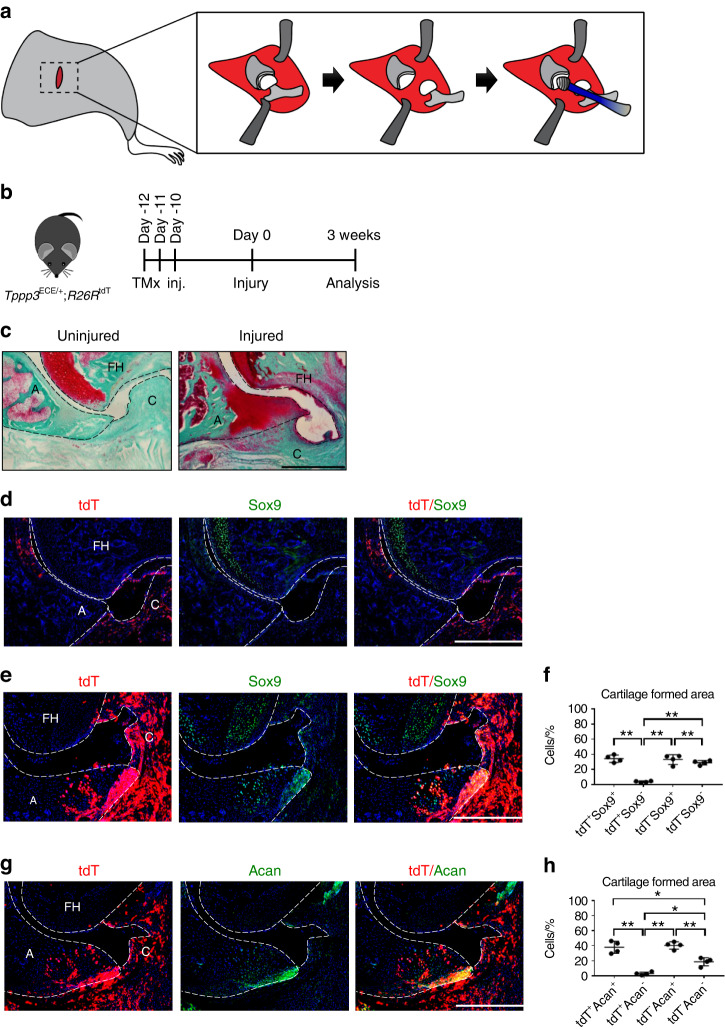
Tppp3^+^ cells in heterotopic cartilage formed an area at an intracapsular site at 3 weeks after HO induction in a hip postarthroplasty HO model. **a** Schematic representation of hip HO induction. **b** *Tppp3*^ECE/+^;R26R^tdT^ animals were administered tamoxifen (TMx) for three d, followed by a 10 d washout period before HO induction. Reporter activity was examined at 3 weeks after injury. **c** The cartilage-formed area was visualized using Saf-O/Fast Green staining at the intracapsular site. Cartilage-like matrix appears red. **d**, **e**, **g** tdT^+^ and Sox9 or Acan immunohistochemical staining within representative transverse sections of hips. The image in (**d**) is the contralateral side, and the images in (**e**, **g**) are the injured side of the hips. A acetabulum, FH femoral head, C capsule. **f**, **h** Quantification of tdT^+^ and Sox9^+^ or Acan^+^ cells within the cartilage-formed area (*n* = 4 animals per group). Scale bars: 500 µm. For all graphs, each dot represents a single animal, with the mean±1 SD indicated. Statistical analysis was performed using one-way ANOVA with Tukey’s post hoc test. ^*^P < 0.05 and ^**^P < 0.01

Given the potential of *Tppp3*^+^ cells to contribute to HO formation in the Achilles tendon, a model of hip postarthroplasty HO was next assessed,^1^ where the cellular and structural context differs. Here, dislocation of the femur and unilateral acetabular reaming were performed, mimicking the initial steps in femoral head arthroplasty, followed by analysis after 3 weeks (Fig. 6a, b). Saf-O/Fast Green staining showed that cartilage tissue (red color) was limited to the articular surface of the femoral head under normal conditions. Upon injury, cartilage was formed in the synovium in the acetabulum area after 3 weeks (Fig. 6c). Next, *Tppp3* reporter activity as well as expression of Sox9 was assessed (Fig. 6d), showing that *Tppp3* expression is restricted to the capsule and to a lesser degree in the acetabulum area. Under control conditions, Sox9 expression was limited to the articular cartilage of the femoral head. After HO induction, *Tppp3*^+^ cells were expanded in number and were noted to express the cartilage markers Sox9 and Acan (Fig. 6e, g). Specifically, *Tppp3-tdT*^+^ cells gave rise to 49.76% and 46.67% of Sox9^+^ and Acan^+^ cells, respectively (Fig. 6f, h).

### *Tppp3*^+^ cells contribute to heterotopic bone in a hip postarthroplasty HO model

**Fig. 7:**
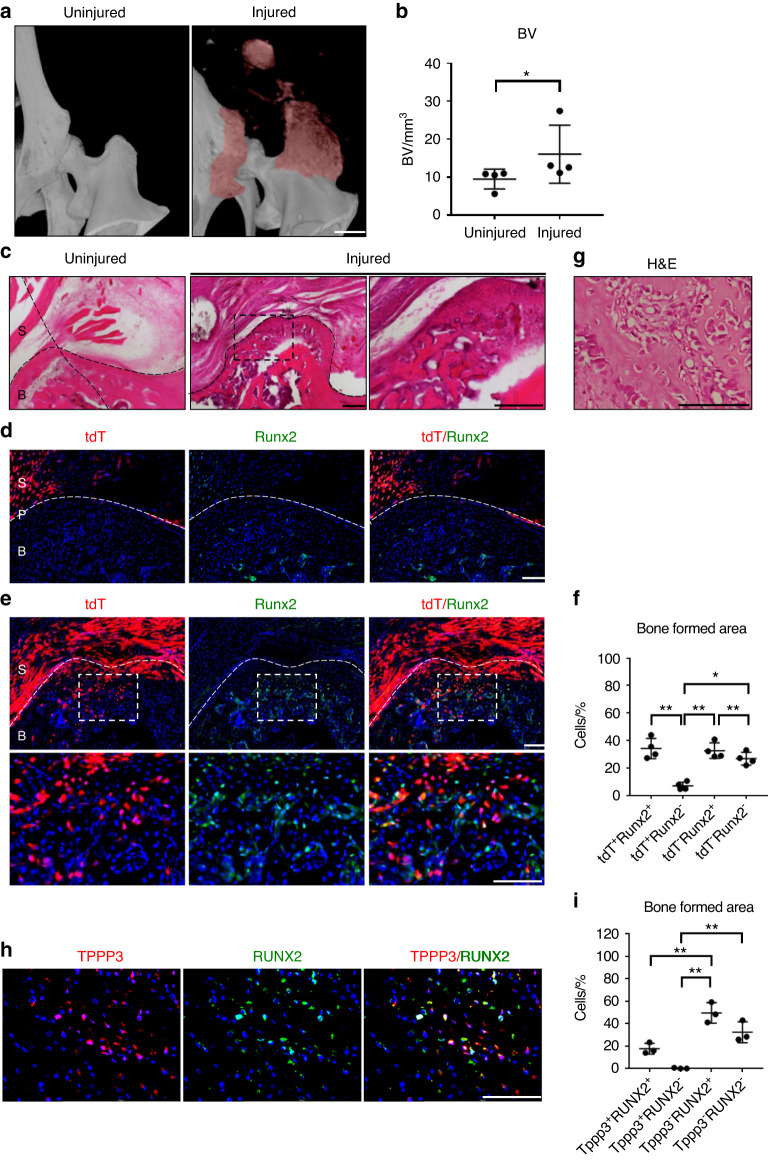
Tppp3^+^ cell contribution to periosteal sites after HO induction in a hip postarthroplasty HO model. **a** µCT reconstruction of bone formation at 3 weeks after injury. Heterotopic bone appears red, while native bone appears white. **b** µCT quantification of heterotopic BV (scale bar: 1 mm, *n* = 4 animals per group). **c** The bone formed area was visualized using H&E staining at the periosteal site of the femoral bone. The dashed black line indicates the margins of heterotopic bone. The dashed black box in the upper panels is magnified in the lower panels (scale bars in panels: 200 µm). **d**, **e** tdT^+^ and Runx2 immunohistochemical staining at periosteal sites of femoral bone (scale bars in panels: 100 µm). The image in (**d**) represents the contralateral control side, and the image in (**e**) represents the injured side. Dashed white lines indicate margins of bone. The dashed white box in the upper panels is magnified in the lower panels. S: synovium; B: bone; P: periosteum. **f** Quantification of tdT^+^ and Runx2^+^ cells within the bone area (*n* = 4 animals per group). **g** H&E staining showed human heterotopic ossification of a human sample (scale bar in panel: 200 µm). **h** TPPP3 and RUNX2 immunofluorescence staining within human heterotopic ossification in a hip joint (scale bar in panel: 200 µm). **i** Quantification of TPPP3^+^ and RUNX2^+^ cells within the bone area (*n* = 3 slides per group). For all graphs, each dot represents a single sample, with the mean±1 SD indicated. Statistical analysis for (**b**) was performed using the Mann-Whitney test. Statistical analysis for (**f**, **i**) was performed using one-way ANOVA with post hoc Tukey’s test. ^*^P < 0.05 and ^**^P < 0.01

Next, the osseous phase of HO formation and *Tppp3* contribution were analyzed in the hip arthroplasty model. First, HO formation was corroborated by high-resolution µCT (Fig. 7a). Indeed, clear aberrant bone formation was found, showing a significant increase of 1.62-fold in volume at the site of injury (injured hip joint; 15.97 mm^3^ and uninjured hip joint; 9.86 mm^3^) (Fig. 7b). H&E staining showed a change in morphology and formation of ectopic bone within the femoral and acetabular periosteum after injury compared to that of the uninjured control (Fig. 7c). Assessment of *Tppp3* tdT reporter activity in uninjured tissue showed no expression in bone tissue, and its expression was restricted to the synovial area (Fig. 7d). After injury, an expansion of *Tppp3-tdT*^+^ cells in the synovium and periosteum was observed, accompanied by the expression of Runx2 in a subset of *Tppp3*-tdT+ cells (Fig. 7e, f). Indeed, *Tppp3-tdT*^*+*^ *cells* were present in 40% of Runx2^+^ osteoblastic cells within the HO site. Further confirmation of this finding was performed by coimmunostaining for Tppp3 and Runx2, in which clear colocalization was observed in the HO site (Fig. S5).

Finally, human pathological samples with aberrant ectopic bone formation were analyzed to investigate the patterns of TPPP3 expression. First, H&E staining was used to identify typical histological features consistent with human HO (Fig. 7g). Next, tissue slides were stained with antibodies against TPPP3 and RUNX2 (Fig. 7h, i), and coexpression of these two markers was determined: TPPP3^*+*^ immunoreactivity was present in 26% of RUNX2^+^ osteoblastic cells within the HO site. Altogether, these data suggest that synovial/tendon sheath progenitor cells expressing Tppp3 acquire an osteoprogenitor phenotype triggered by traumatic injury, resulting in aberrant stem cell differentiation in soft tissues. Moreover, the contribution of *Tppp3*^*+*^ cells seems to be conserved across anatomical sites of HO and found in both mouse and human tissues.

## Discussion

Traumatic HO is the result of an aberrant repair process in which mesenchymal stem cells differentiate into chondrocytes and osteoblasts that form bone, replacing the native soft tissue.^1^ The formation of ectopic bone involves a complex network of interactions between progenitor cells, immune cells, blood vessels and even nerves.^8,20,23^ Although mesenchymal cells are known to contribute to HO, the specific subsets that participate in this aberrant process are poorly understood. Here, we determined to what extent synovial/tendon sheath progenitor cells expressing *Tppp3* contribute to HO formation. Upon injury, these cells proliferate, contributing to tissue remodeling, and undergo the typical HO developmental sequence of cartilage formation and subsequent heterotopic bone formation. Moreover, scRNA-seq analysis identified Tppp3 (expressed in mesenchymal progenitor cells and pericytes) as an early progenitor of bone by trajectory analysis. Overall, this study described a newly defined population of tendon progenitors that contribute to ectopic bone formation in vivo, which may have therapeutic implications for preventing HO formation.

Previous studies have shown the contribution of mesenchymal cells to HO formation using tamoxifen-inducible mice for *Gli1, Scx*, and *Pdgfra*.^10,14,19^ Nonetheless, the expression of these markers is observed in differentiated cells such as Scx^+^ tenocytes or in a wide variety of cell types such as Pdgfra^+^ mesenchymal cells. However, *Tppp3* expression was restricted to the synovium/tendon sheath, representing a primitive progenitor rarely expressed by tenocytes. Indeed, trajectory analyses showed that *Tppp3*^+^ *Pdgfra*^+^ cells along with CD146^+^ cells (pericytes) were early in pseudotime, merging into a common branch toward osteochondral differentiation, losing the expression of *Tppp3*. Moreover, stratification of the trajectory into timepoints showed that *Tppp3* is expressed only in undifferentiated cells. In addition, mesenchymal cells can promote HO formation by the release of soluble molecules.^8^ Our group has previously shown that *Vegfa* released by mesenchymal cells is crucial for heterotopic ossification,^24^ which correlated with the most undifferentiated cell cluster expressing *Tppp3*. These cells express not only *Vegfa* but also *Bmp4*, *Bmp7* and *Fgf2*, among others, suggesting a regulatory effect of undifferentiated *Tppp3*^+^ cells, which may promote other subsets to undergo ossification. In addition, analysis of factors that stimulate tendon differentiation were likewise expressed among Tppp3^+^ cells, such as Bmp family members and Tgfb isoforms.^22^

An interesting observation in this study was the clear difference in the contribution of *Tppp3*^+^ cells to HO in our two injury models. The synovial membrane of the hip contains *Tppp3*^+^ progenitor cells that comprise approximately 40%–50% of the total aberrant bone area, while in the Achilles tendon, this represents approximately 20% of total bone cells. One reason for this may be the phenotype of the cells from distinct origins. For instance, in the Achilles tendon, the cells that acquire an aberrant stem cell phenotype are tendon progenitors that must undergo a complex process of endochondral ossification.^4^ In contrast, cartilage progenitor cells in the hip model are perhaps more primed to become osteoblasts, which is likely the result of bypassing certain stages of endochondral ossification due to their cartilaginous nature when compared to tendons. This finding is in line with studies examining the transition of chondrocytes to osteoblasts and osteocytes during endochondral bone formation.^25^ Moreover, the tissue-specific stroma and cartilage in the hip may release soluble molecules that further promote the expansion and differentiation of *Tppp3*^+^ cells. Nonetheless, more studies are needed to fully understand how *Tppp3*^+^ progenitor cells in different anatomical depots behave.

This study has several limitations for consideration. First, our scRNA-seq data did not contain a reporter gene that can help trace the differentiation trajectory of cells. Second, the contribution and regulation of other cellular components, such as immune cells, blood vessels and nerves, were not assessed. For instance, previous reports from our group showed that HO is driven by coordinated growth of blood vessels and nerves.^24^ Notably, in our scRNA-seq data, blood vessel-associated pericytes and nerve-associated Schwann cells both showed *Tppp3* expression, although these are both small cell populations in our models. In this context, there is no information on the effect or interaction of Tppp3^+^ cells in the accessory regulators of HO formation. Moreover, CD146^+^ cells have been described as tendon progenitors expressing Tppp3,^26^ which may indicate a more complex process regulating angiogenesis. Finally, the Tppp3 lineage contribution was dependent on the anatomical site, which may indicate a mechanical and functional component as well as a progenitor phenotype with tissue specificity that dictates their capability to differentiate.

In conclusion, this study provides evidence of the contribution and regulatory potential of synovial/tendon sheath progenitor *Tppp3*^+^ cells to form HO across traumatic injury models and the conserved cell identity in human HO samples. These novel observations have implications for the mechanisms that drive aberrant stem cell differentiation in soft tissues and possibly for the development and testing of new therapeutic strategies.

## Materials and methods

### Mice

All animal procedures were approved by the Institutional Animal Care and Use Committee (IACUC) of Johns Hopkins University (MO19M366). All animals were housed at 18–22 °C, 50% (± 20%), and a 12 h light-dark cycle with ad libitum access to food and water in IACUC-supervised facilities. Mouse strains were made in-house or were sourced from the Jackson Laboratory or Fan Laboratory (Table S1). *Tppp3*^ECE/+^;*R26R*^tdT^ mice were obtained by crossing *Tppp3*^ECE^ mice with *R26R*^tdT^ mice. *Tppp3*^ECE/+^;*R26R*^tdT^;*Pdgfra*^H2B-eGFP^ mice were obtained by crossing *Tppp3*^ECE/+^;*R26R*^tdT^ mice with *Pdgfra*^H2B-eGFP^ mice. *Tppp3*^ECE/+^;*R26R*^tdT^;*Scx*-GFP mice were obtained by crossing *Tppp3*^ECE/+^;*R26R*^tdT^ mice with *Scx*-GFP. Data from mixed-sex groups were used. Mice were used at 8 weeks of age.

### Tamoxifen administration

Tamoxifen (TMX; T5648, Sigma-Aldrich, St. Louis, MO) was prepared as a 20 mg·mL^−1^ stock in sunflower oil and administered by intraperitoneal (i.p.) injection at 10 µL per g body weight for three consecutive d, followed by a 10 d chase period.

### Mouse HO models

Animals were anesthetized with inhaled isoflurane (NDC 66794-07-25, Piramal Enterprises, Limited. Telangana, India) delivered with combined oxygen and nitrous oxide (1.5:2 ratio). The leg or hip was prepared for surgery; hair was closely clipped, and skin was disinfected with betadine solution (DNC 67618-155-16, Purdue products L.P., Stamford, Ct, USA).

For the Achilles tendon HO model,^20,27^ a 1 cm skin incision was made directly over the left calf to expose the Achilles from its origin on the distal end of the gastrocnemius to the insertion at the calcaneus. The Achilles tendon was completely transected at its midpoint. The injured area was washed with saline, and the skin was sutured with 5-0 Vicryl suture (J463G, Ethicon, Cincinnati, OH, USA). For a partial thickness burn, hair on the mouse dorsum was removed, and a burn was made by placing a metal brand heated to 60 °C in a water bath against the exposed skin (30% total body surface area) for 18 s (Fig. 1a). These mice were sacrificed at 0, 1, 3 and 9 weeks after injury for analysis (Fig. 1b). For the hip postarthroplasty HO model,^1^ the right leg was operated on in all cases. A 2 cm skin incision was made centered on the greater trochanter and directed proximal to the iliac crest and distally over the lateral shaft of the femur. The joint was reached following the intermuscular plane between the rectus femoris and gluteus medius muscles. Then, a capsulotomy was performed. The femoral head was partially dislocated to give access to the acetabular reamer, and the articular cartilage was removed using a reamer 1.1.2 mm in diameter (Cell-point Scientific, MD, USA) and a micropower drill (Roboz Surgical Instrument Co., MD, USA). The surgical site was washed with saline solution, and the skin was closed with 5-0 Vicryl sutures (J463G, Ethicon, Cincinnati, OH, USA). Samples were collected at 3 weeks post-injury for analysis.

#### Characterization of Tppp3^+^ cells derived from the Achilles tendon

Achilles tendons from *n* = 3 8-wk-old male *Tppp3*^ECE/+^;*R26R*^tdT^;*Scx*-GFP mice were dissected and dissociated in 3 mg·mL^−1^ Collagen I (Worthington) and 4 mg·mL^−1^ dispase II (Roche) in PBS for 1.5 h at 180 r·min^−^^1^ at 37 °C.^20^ The tissues were then lightly triturated with sterilized Pasteur pipettes (VWR), run through a 40 μm filter (VWR) and washed with 3 mL of αMEM (Gibco) with 20% FBS. Filtered cells were centrifuged at 300 × *g* for 30 min at 4 °C. The cell pellet was washed twice with 1 mL of PBS, and the cells were resuspended in FACS buffer (1% BSA, 1 mmol·L^−1^ EDTA in PBS). Cell concentration and viability (by Trypan blue exclusion) were confirmed using a Countess II Automated Cell Counter (Thermo Fisher Scientific) and hemocytometer (VWR). Cells were distinguished by forward and side scatter, and sorting was gated using FITC-A (GFP) and PE-A (tdT) filters. The isolated Tppp3^+^ cells were initially expanded in growth medium (DMEM + 20% FBS + 1% penicillin‒streptomycin (Gibco)). At confluence, the cells were trypsinized and seeded for subsequent differentiation. Cells grown in growth medium served as undifferentiated controls for each assay.

For adipogenic differentiation, cells were seeded at a density of 2 × 10^5^ cells per well. At 80% confluence, the growth medium was replaced by murine adipogenic induction medium (MesenCult adipogenic differentiation kit, Stemcell Technologies). At d14 of differentiation, both groups were stained with Oil Red O (Sigma-Aldrich). Images of oil vacuole deposition were captured under a bright field using a Leica DM ICC50W microscope.

For osteogenic differentiation, at confluence, the cells were switched to osteogenic medium consisting of DMEM, 10% FBS, and 1% penicillin-streptomycin with 10 mmol·L^−1^ β-glycerophosphate, 50 μmol·L^−1^ ascorbic acid, and 1 mmol·L^−1^ dexamethasone. At d14, osteogenesis was detected by staining the cells with 2% alizarin red staining solution (Sigma-Aldrich, pH 4.2) for calcium nodules to stain orange red. Images were captured by a Leica DM ICC50W microscope.

For chondrogenic differentiation, confluent *Tppp3*^*+*^ cells were changed to chondrogenic induction medium (Mesencult-ACF chondrogenic differentiation kit, Stemcell Technologies). At d21, the differentiated cells and controls were stained with Alcian blue 8GX (Sigma-Aldrich). Chondrogenesis was confirmed by blue color staining, and images were taken by a Leica DM ICC50W microscope.

For tenogenic differentiation of *Tppp3*^*+*^ cells, tenogenic differentiation medium consisting of aMEM, 10% FBS, 1% penicillin-streptomycin, 50 μg·mL^−1^ ascorbic acid and 10 ng·mL^−1^ TGFβ-1 was used. At d14 of differentiation, images of Scleraxis-GFP reporter activity were captured by a Zeiss LSM 800 confocal microscope, and images were processed with ZEN blue software (Zeiss, USA).

### Human samples

Human hip HO specimens were identified in our surgical pathology archives in the form of formalin-fixed, paraffin-embedded blocks, and the use of patient pathology specimens complied with all relevant ethical regulations. All specimens were coded to protect the confidentiality of personal information and obtained under Johns Hopkins University Institutional Review Board approval with informed consent (IRB number; NA_00028453). All specimens were reviewed by a pathologist to verify the diagnostic accuracy (A.W.J.).

### Histology and immunohistochemistry

Mouse tissues were harvested and fixed in 4% paraformaldehyde at 4 °C for 24 h. Tissues were washed with PBS for 1 h and decalcified in 14% EDTA (Sigma-Aldrich) for 21 d at 4 °C. For cryosections, tissues were cryoprotected in 30% sucrose overnight at 4 °C before embedding in OCT (Tissue-Tek 4583, Torrance, CA). The Achilles tendon was sectioned at 12 μm or 30 µm thickness. Sections (12 µm) were used for routine Safranin-O/Fast green (Saf-O/Fast green)^28^ and hematoxylin and eosin (H&E)^29,30^ staining and immunohistochemistry. For detection of fluorescent reporter proteins, 30 µm slides were washed in PBS 3x for 10 min and then mounted with DAPI mounting solution (Vectashield H-1500, Vector Laboratories, Burlingame, CA).

For immunohistochemistry, slides were washed in PBS 3x for 10 min and permeabilized with 0.5% Triton X for 30 min. Subsequently, 5% normal goat serum was applied for 1 h and incubated in primary antibodies overnight at 4 °C in a humidified chamber. Next, the slides were washed in PBS, incubated in 3% normal goat serum with secondary antibody for 1 h at 25 °C and then mounted with DAPI mounting solution. Images were taken using upright light and fluorescence microscopy (Leica DM6, Leica Microsystems, Inc., Buffalo Grove. IL). Images were processed using ImageJ software (National Institutes of Health, MD, USA).

For human samples, slides were dewaxed with xylene and hydrated by immersion in decreasing concentrations of ethanol. Slides were used for Saf-O/Fast Green and H&E staining. For immunohistochemistry, antigen retrieval was performed after dewaxing and hydration. Briefly, slides were boiled in 10 mmol·L^−1^ citrate buffer, pH 6, for 20 min, followed by immunostaining as described above. For detailed antibody information, see Table S2.

### Micro computed tomography (µCT) imaging and analysis

Mouse hindlimbs and hips were harvested and imaged at 9 weeks and 3 weeks postinjury, respectively, using a Skyscan 1275 scanner (Bruker MicroCT, Kontich, Belgium) with the following settings: 65 kV, 153 µA, 1 mm aluminum filter in 180°, 6 frames per 0.3° with a 10 µm voxel size. Images were reconstructed using NRecon and exported as DICOM files. DataViewer software was used to realign the images, and quantitative parameters were assessed using Skyscan- CTan software (SkyScan, Kontich, Belgium). Cylindrical regions of interest (ROI) of 3.5 mm in diameter and 3 mm in thickness were used for Achilles tendon-associated HO samples. For hip postarthroplasty HO samples, cylindrical regions of interest (ROI) of 4.5 mm in diameter and 3 mm in thickness were used. A threshold value range of 80–255 was used. After global thresholding was carried out, a 3-dimensional (3D) data analysis including bone volume (BV) was performed. Three-dimensional reconstructions were performed using Dragonfly (ORS, Inc., Montreal, Canada).

#### Single-cell RNA sequencing (scRNA-seq) analysis

scRNA-seq data were obtained from a published dataset from our group (accession number: GSE126060), in which the tendon injury model of HO^20^ was examined at 0, 7 and 21 d after injury. *n* = 3 mice per timepoint were used (6–10 weeks male C57BL/6 J mice), which included a total of 10 119 cells (3 815 from 0 d, 3 144 from 7 d, and 3 160 from 21 d). Downstream analysis steps were performed using Seurat as previously described.^24,31^ Briefly, cells with fewer than 500 genes, with more than 60 000 UMIs, or expressing a fraction of mitochondrial UMIs higher than 0.1 were filtered for quality control. Genes present in fewer than 3 cells per set were discarded. Unsupervised clustering (Louvain algorithm) was used to identify cell populations. Trajectory analysis was performed using Monocle2.^32^ DAVID Bioinformatics was used for enrichment analyses.^33^

### Statistics

Quantitative data are expressed as the mean±1 SD with individual datapoints shown. The number of samples is shown in the figure legends. A Shapiro‒Wilk test for normality was performed for all datasets. Parametric data were analyzed using two-sided Student’s *t* test when comparing two groups or one-way ANOVA with post hoc Dunn’s multiple comparisons test when comparing more than two groups. Nonparametric data were analyzed with a Mann-Whitney U test when comparing two groups or a Kruskal‒Wallis one-way analysis with post hoc Tukey’s test when comparing more than two groups. All statistical analyses were performed with GraphPad Software 8.1 (GraphPad Software, San Diego, California, USA). ^*^P < 0.05 and ^**^P < 0.01 were considered significant.

## Supplementary information

Supplemental_material
