# Supplementary material for: Tppp3+ synovial/tendon sheath progenitor cells contribute to heterotopic bone after trauma
Source: Bone Res. 2023 Jul 21;11:39. doi: 10.1038/s41413-023-00272-x (PMC10361999; doi:10.1038/s41413-023-00272-x)
Supplement: Supplementary file 1 — Supplemental_material [file 41413_2023_272_MOESM1_ESM.docx]

**Supplementary Table 1 | Mouse alleles used.**

| **Mouse allele cited in text** | **Allele full name** | **Supplier** | **Stock number** | **Citation** | **Pubmed ID** |
| --- | --- | --- | --- | --- | --- |
| C57BL/6J | C57BL/6J | JAX | 000664 | - |  |
| *Pdgfra*^H2B-eGFP^ | B6.129S4-*Pdgfra^tm11(EGFP)Sor^*/J | JAX | 007669 | ^40^Hamilton et al.(2003) *Mol Cell Biol* **23**(11): 4013-25 | 12748302 |
| *R26R*^tdT^ | B6.Cg-*Gt(ROSA)26Sor^tm14(CAG-tdTomato)Hze^*/J | JAX | 007908 | ^15^Madisen et al. (2010) *Nat Neurosci* **13**(1):133-40 | 20023653 |
| Scx-GFP | tg(ScxGFP) | Donated by the Fan laboratory | - | ^31^Pryce et al. (2007) *Dev Dyn* **236**(6):1677-82. | 17497702 |
| *Tppp3*^ECE^ | *Tppp3*^P2A-ERT2CreERT2^ | Donated by  The Fan laboratory | - | ^1^Harvey et a. (2019) *Nat Cell Biol* **21**(12):1490-503 | 31768046 |

**Supplementary Table 2 | Antibodies used.**

| **Antibody** | **Catalog number (Company)** | **Dilution** |
| --- | --- | --- |
| rabbit anti-Aggrecan (Acan) | ab216965 (Abcam, Cambridge, UK) | 1:100 |
| rabbit anti-Osteocalcin (OCN) | ab93876 (Abcam) | 1:100 |
| rabbit anti-Runt-related transcription factor 2 (Runx2) | ab192256 (Abcam) | 1:100 |
| mouse anti-Runx2 | ab76956 (Abcam) | 1:100 |
| rabbit anti-SRY-Box Transcription Factor 9 (Sox9) | ab185230 (Abcam) | 1:100 |
| rabbit anti-Tenomodulin (Tnmd) | ab203676 (Abcam) | 1:100 |
| rabbit anti-Tppp3 | ab150998 (Abcam) | 1:100 |
| goat anti-rabbit (Alexa Fluor 488) | ab150077 (Abcam) | 1:1,000 |
| goat anti-mouse (Alexa Fluor 594) | ab150116 (Abcam) | 1:1,000 |


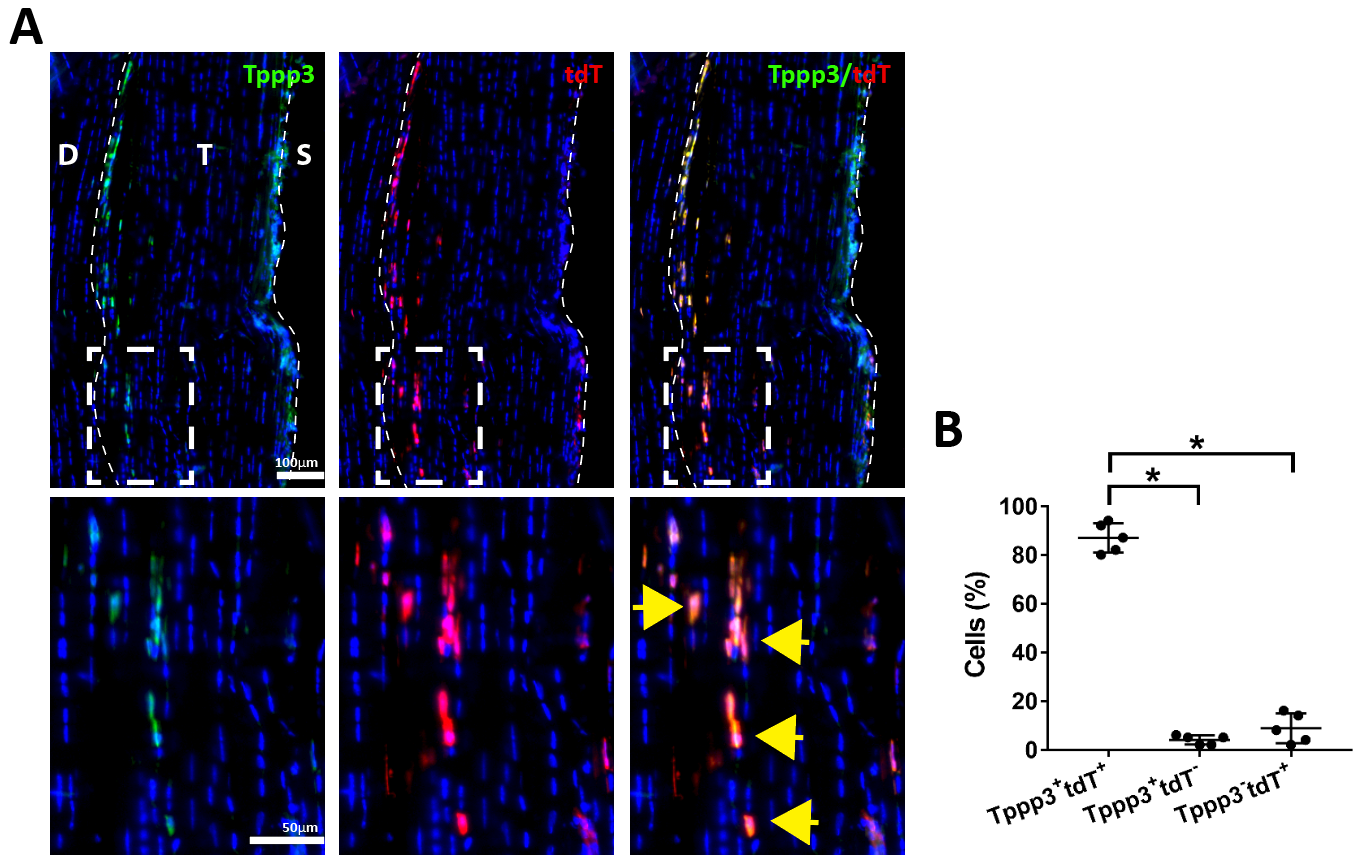


**Supplementary Figure S1**. Tppp3 expression in *tdT^+^* cells within the uninjured Achilles tendon in *Tppp3*^ECE/+^;R26R^tdT^ mice. Images obtained 10 d after Tamoxifen labeling. (A) Tppp3 immunohistochemical staining with representative sagittal sections of the distal tenotomy site. Yellow arrows indicate cells with both Tppp3*^+^* and *tdT^+^* signals. Dashed white line indicate margins of the Achilles tendon and dashed white box in upper panels are magnified in lower panels. D: deep; T: tendon; S: superficial, (B) Quantification of Tppp3*^+^* and *tdT^+^* cells and their level of co-expression within the tendon sheath. For graphs, each dot represents a single animal, with mean ± 1 SD indicated. N=5 mice. Statistical analysis was performed using one-way ANOVA with post hoc Tukey’s test. **P*<0.05.


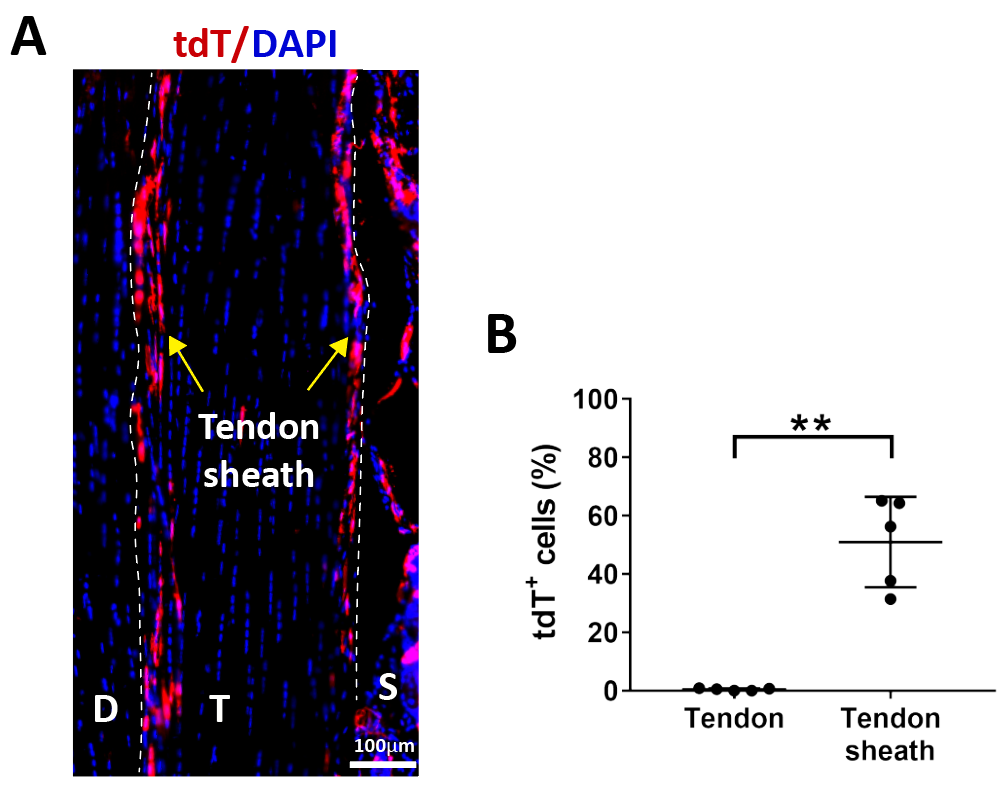


**Supplementary Figure S2**. Distribution of *Tppp3*^+^ cells within tendon and tendon sheath area in the uninjured Achilles tendon in *Tppp3*^ECE/+^;R26R^tdT^ mice. Images taken 10 d after tamoxifen injection. (A) *tdT^+^* cells were visualized using sagittal sections of the Achilles tendon. Dashed white lines indicate outer margins of the Achilles tendon sheath. D: deep; T: tendon; S: superficial, (B) Quantification of *tdT^+^* cells within the tendon and tendon sheath area. N=5 mice. For all graphs, each dot represents a single animal, with mean ± 1 SD indicated. Statistical analysis was performed using one-way ANOVA with post hoc Tukey’s test. ***P*<0.01.


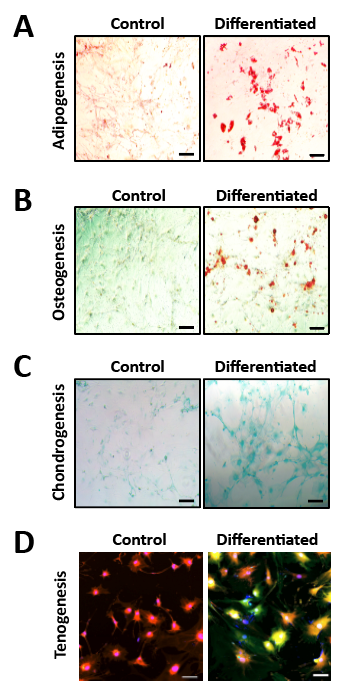

**Supplementary Figure S3.** Multilineage differentiation potential of FACS-isolated Achilles tendon *Tppp3-tdT^+^* cells. (**A**) Adipogenic differentiation. Bright field image at d14 in differentiated as compared to undifferentiated cells by Oil red O staining. (**B**) Osteogenic differentiation of *Tppp3^+^* cells as compared to undifferentiated cells at d14 by Alizarin red staining. (**C**) Chondrogenesis of *Tppp3^+^* cells as compared to undifferentiated control at d21 by Alcian blue staining. (**D**) Tenogenic differentiation of *Tppp3^+^* cells, by expression of Scleraxis-GFP (green, right) by confocal imaging at d 14. Both cells appear tdT^+^. Scale bar: 100μm. N=3 technical replicates for each group.


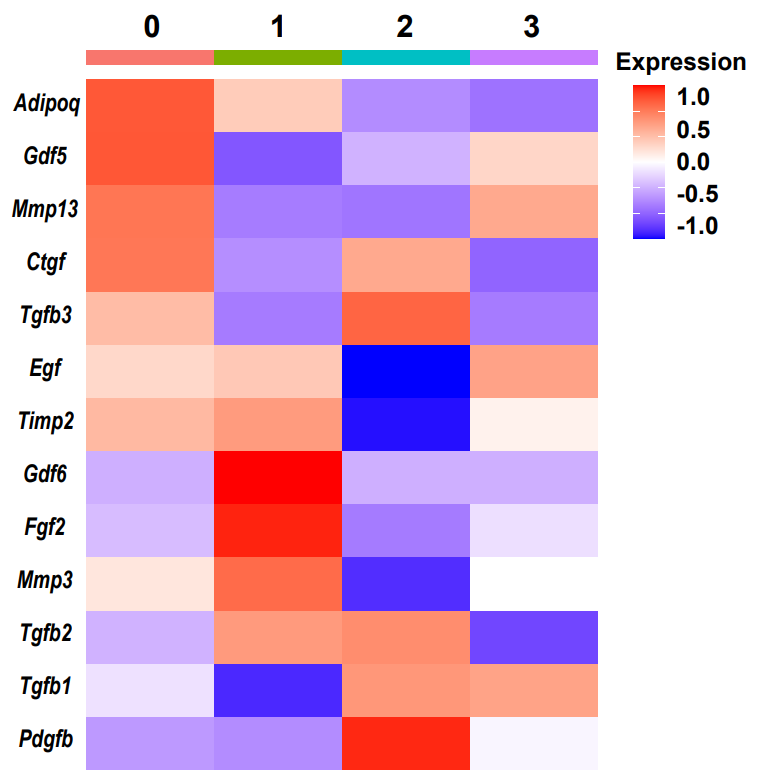


**Supplementary Figure S4.** Gene Expression of molecules promoting tenogenic differentiation across cell mesenchymal lineage clusters.


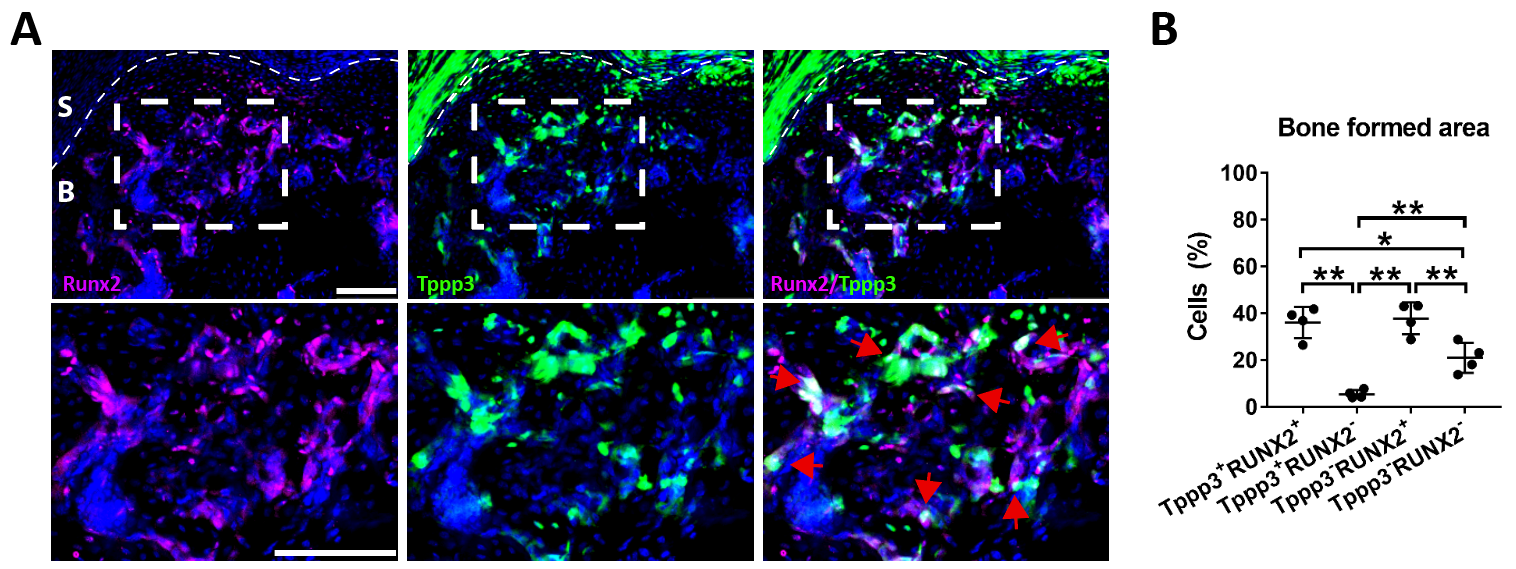


**Supplementary Figure S5.** Tppp3 and Runx2 are co-expressed after HO formation in a mouse hip post-arthroplasty model. (A) Tppp3 and Runx2 immunofluorescent staining within the HO site at 3 weeks post-injury. Red arrows indicate the cells with co-expression of Runx2 and Tppp3. Dashed white line indicate margins of the heterotopic bone and dashed white box in upper panels is magnified in lower panels. S: synovium; B: bone. Scale bar: 200 µm. (B) Quantification of Tppp3^+^ and Runx2^+^ cells within the bone area (N=4 animals per group). For all graphs, each dot represents a single animal, with mean ± 1 SD indicated. Statistical analysis was performed using one-way ANOVA with post hoc Tukey’s test. **P*<0.05 and ***P*<0.01.
